# De-extinction: a novel and remarkable case of bio-objectification

**DOI:** 10.3325/cmj.2014.55.423

**Published:** 2014-08

**Authors:** Lucia Martinelli, Markku Oksanen, Helena Siipi

**Affiliations:** 1 MUSE – Science Museum, Trento, Italy lucia.martinelli@muse.it; 2 Department of Behavioural Sciences and Philosophy, University of Turku, Turku, Finland majuok@utu.fi; 3 Department of Behavioural Sciences and Philosophy, University of Turku, Turku, Finland helsii@utu.fi

De-extinction is the process of creating an organism which is – or greatly resembles – a member of an extinct species (1). Contemporary biotechnology offers various promising alternatives for achieving this purpose, including the techniques that have already been applied to preserving endangered species (2). De-extinction requires an in-depth study of the biophysical conditions where the species can live and reproduce in relation to other species – including humans – and adapt to the environmental changes. In any case, risk and harm evaluation on the impact of the “re-birth” of species is necessary. There is a number of crucial ethical issues concerning de-extinction. They include the meanings of concepts such as “nature,” “species,” “evolution,” “biodiversity,” “death,” and “wildlife” in relation to human behavior and human impact on nature (3). In 2013, de-extinction became popular through press and public events; the *National Geographic* devoted a cover story to this topic and presented various possibilities and scenarios about the most suitable candidates. The Revive & Restore network, with the support of TED and in partnership with the National Geographic Society, convened the TEDxDeExtinction conference bringing together conservationists, genetic technology practitioners, scientists, and ethicists (*http://www.ted.com/tedx/events/7650*). Because the revival of extinct animals inspires imagination (4), de-extinction has been a topic of science fiction novels, such as John Brosnan’s Carnosaur (1984) and Michael Crichton’s Jurassic Park (1990) and their film adaptations.

Following a systematic philosophical and ethical analysis on animal de-extinction in the context of ecological restoration (3), this article analyzes de-extinction from the standpoint of bio-objectification and considers how de-extinction is a case of bio-objectification.

## DE-EXTINCTION

The use of gene-technology for species conservation is an emerging and controversial topic: while some are reluctant to accept or contemplate the appropriateness of technological “hands on” approaches, numerous research groups around the world are already developing these techniques not only for conserving but also for bringing back to life extinct species. A milestone in endangered species preservation was the production of American chestnut trees with enhanced resistance to the blight fungus (*Cryphonectria parasitica* [Murr.] Barr.) that causes chestnut blight. They were generated with gene transfer techniques following previous unsuccessful efforts based on crossings and back-crossings between the American chestnuts (*Castanea dentata* [Marsh.] Borkh.) and the blight-resistant Chinese chestnuts (*Castanea mollissima*) (5). As for de-extinction, attention seems, however, to be more addressed to animals than to plants, and efforts have already been invested in such iconic animal species as the woolly mammoth, the moa, the Carolina parakeet, the Yangtse river dolphin, and the passenger pigeon, or peculiar creatures like the gastric brooding frogs. If the research groups working toward de-extinction succeed – and many think that they soon will (6-8) – their achievements would challenge the perceived irreversibility of extinction.

Being aware that de-extinction will in foreseeable future become an established conservation strategy, the International Union for the Conservation of Nature (IUCN) published the Guidelines on Reintroduction and Other Conservation Translocations with a list of 20 selected faunal “most suitable candidates” and recommendations for risk evaluation (9). On this list, the most numerous are mammalians, followed by birds and an insect (Xerces blue butterﬂy).

“De-extinction” as an expression was coined in 2012 to denote the goals and the reversal of extinction. The first Wikipedia entry is dated June 7, 2013 and no other entries are yet available (as of June 2014) in major dictionaries. Terminology for this new and emerging field of research and its prospective outcomes is not fully established. Along “de-extinction” terms like “extinction reversal,” “re-creation,” “resurrection,” “reviving,” and “resuscitation” are widely used. As a result, discussing de-extinction implies taking part in constructing a “science of resurrection” (10,11).

The most promising strategies for achieving de-extinction are back-breeding, cross-species cloning, and genetic engineering (12). Back-breeding is a selective breeding from individual organisms genetically and morphologically close enough to the extinct species. The aim is to bring back the qualities lost in extinction and in this way produce species as similar as possible to the extinct one. It is impossible to turn the evolutionary clock backward, but there are cases that might be characterized as successful. In the 1920s and 1930s, the German brothers Lutz and Heinz Heck had a plan of reviving two extinct species, the aurochs (*Bos primigenius*) and the tarpan (*Equus ferus ferus*), the wild ancestors of the cow and the horse. The population they produced, and which still exists, differs from the “ordinary” cows and horses but, most fairly considered, these animals are only lookalikes of their extinct “ancestors” (13,14). Their plan, however, inspired a more recent project in South-Africa to revive the quagga (*Equus quagga quagga*). It is a subspecies of the common zebra that went extinct in the late 19th century and only pictures and a few taxidermied samples are left in natural history museums. In the Quagga Project, the aim is to back-breed a population morphologically close to the “original,” while no other species-specific features are pursued (*http://www.quaggaproject.org*/). The project was launched in 1987 and by 2005 fifth generation animals had recognizable quagga features.

Cross-species cloning and genetic engineering rely on advanced gene technologies such as cloning, DNA synthesizing, and genome reconstruction (7). In addition to technological development, tissues from extinct species are needed. Repositories for biodiversity are a good source for the necessary material, as well as ancient DNA from carcasses of long extinct animals (1). An impaired or contaminated (ancient) genome may have to be “repaired” or “perfected” by genetic engineering. In case of animals, the next step is to create live individuals by cross-species cloning followed by back-breeding of the lineage toward the extinct ancestor. The woolly mammoth (*Mammuthus primigenius*), for instance, could be born from elephant egg cell and elephant surrogate mother and after that selective breeding methods could be used to diminish its elephant features and enhance its “mammothness.”

The de-extinction projects are controversial, and many concerns about animal welfare and environmental impacts of (re)introduced species have been raised (7,15). Some critics worry that the success of de-extinction technology might diminish the desire to protect species (12,15-17). Also the ambiguous species identity of animals and other hybridized organisms born from the de-extinction procedures have gained attention (7,16-18).

## RE-CREATED ORGANISMS AS BIO-OBJECTS

De-extinction is a novel case for illustrating the process of “bio-objectification,” ie, “process by which life is made an object by human beings” (19). De-extinction can be taken as an instance of bio-objectification because the wild species, its preserved tissues, or produced organisms are turned into objects used for human purposes, from knowledge enhancement, species conservation, and scientific discoveries to entertainment in zoos and exhibits.

The organisms born from de-extinction procedures are bio-objects, since they are “living beings […], and yet at the same time […] objects that can be used, controlled, and traded for human purposes” (19). These organisms carry various crucial features we attribute to bio-objects. As “boundary crawlers” (20), they cross borders between natural and artificial, death and life; extinction and still existing; past and present. Moreover, questions rise concerning their identity (7,21) since live specimens of extinct species may not be quite the original species; and if so, what kinds of techno-social processes would determine their identity? For all these peculiarities, re-created organisms definitely appear to be “out of place,” a feature often associated with bio-objects (22,23). This feature is most obvious in re-created organisms that live their whole life “out of nature” in zoos, laboratories, or even in touring exhibitions. Yet, for many re-created organisms this may be the only feasible possibility. Ecosystems are constantly evolving and changing, and it may be difficult to find suitable niches for the re-created organisms (24). For many extinct species, (human caused) changes in the living environment were the cause for extinction, and their native range can no longer provide them a suitable living environment. Thus, we can say that also in their native range, they would be out of place in the sense of their environment being unsuitable for them. Thus, re-creating these kinds of organism destines them to be out of place. However, if extinct species would be adaptive and able to modify a habitat to become suitable to them, as suggested for the woolly mammoth in Siberia (25), it would be possible to see de-extinction as a process that can contribute to putting species back into their places.

## THE FINALITY OF EXTINCTION

To recognize the bio-objectification processes taking place in de-extinction, the issue of extinction should be also analyzed. The common meaning of extinction points to the end and the finality of something that has existed. In Collins Concise Dictionary “extinction” is a noun denoting “complete destruction” and “annihilation,” and “extinct” is an adjective referring to an animal or plant species that has “died out.” Thus, according to the standard definition, extinction is “final.” The philosopher Alastair S. Gunn (26) explains the meaning of extinction as follows: “…extinction says something about the future of the class – that once it becomes a null class, it can never come to have members again.” Granted that the moment of extinction is associated with the death of the last individual of a species, Gunn’s view implies that in its literal sense de-extinction is never possible, and organisms that have come into existence through de-extinction procedures cannot be representatives of the species that once died out. Rather, they would be living artifacts and members of a new human-made species, ie, bio-objects, in our understanding. As a result, the process of bio-objectification should in their case be seen similar to those synthetic biology projects that design and construct new biological organisms that do not exist in nature (for definitions of synthetic biology, see *http://www.synbioproject.org/topics/synbio101/definition/*). In both cases, the individuals fall into the same category of *de novo* species and not into some previously existing category. Even though the features of the organisms born through de-extinction procedures resemble the features found in representatives of an extinct species, they are distinct from them and first and foremost bio-objects created by human beings.

The research groups working toward the goal of de-extinction usually do not conceptualize their work as a creation of new types of beings. Rather, the US-based Revive and Restore network (*http://longnow.org/revive/what-we-do/)* describes their aim as “genetic *rescue* for endangered and extinct species” (italics added). Similarly, the Quagga Project identifies its work as an attempt to “*bring back* animals from extinction” (italics added). A presupposition behind this research seems to be that even though extinction has this far been final, the technological development can in near future make it possible to undo extinction. As a matter of fact, many see as the ultimate achievement of the research and development in question that “extinction need not be forever” (27) and that “extinction is not forever” (28). Under this kind of understanding of extinction and de-extinction, the bio-objectification can be seen as a process in which a species (or at least individuals belonging to it) are taken under human control and manipulation. Even though the organisms created do not (at least necessarily) represent new species, their existence is human-dependent and human-controlled and to a great extent inspired by human interests related to them.

During the history of life on Earth, countless plant species have disappeared and they are commonly regarded as extinct. However, when seeds from a plant species are preserved in cryobanks or in natural conditions as in the (ant)arctic permafrost, or in the “global seed vault” in Svalbard (*http://www.croptrust.org/content/svalbard-global-seed-vault*), it is anticipated that this frozen biological material can germinate. These seed banks were constructed upon this presumption but it was not clarified under which conditions such entities with “suspended life” could resume living. Paleobotanists and horticulturalists are competing who will germinate the oldest plant material. Currently, the record is held by a Russian group that has succeeded in regenerating and micropropagating fertile plants from 30 000 old fruits of *Silene stenophylla* Ledeb. preserved in the Siberian permafrost (29). Also, there are examples of highly resilient animal species of which tardigrade (*Tardigrada*) and brine shrimp (*Artemia salina*) are the best known (30). In unfavorable conditions, tardigrades remain in “cryptobiosis,” a sort of dormancy, and are capable of “resume living” even after a hundred years. Also the thick-shelled eggs of artemia can stay in dormancy for long periods of time (30). As these examples indicate, the lure of regenerating ancient seeds and their commercial utilization is strong, since ancient specimens would be attractive “trophy plants” to be shown in gardens, greenhouses, and other exhibitions.

## PERSPECTIVES OF DE-EXTINCTION

The de-extinction experiments enhance technical knowledge about DNA manipulation. When based on animal cloning, they propose new insights into important questions related to genetics and embryo development, primarily the complex relations between nucleus and cytoplasm, and the relevance of epigenetics (6). Moreover, de-extinction opens interesting perspectives for bio-medical applications, as in the case of the gastric brooding frogs (*Rheobatrachus silus*), which became extinct in the mid-1980s (31). The unique ability of these frogs to change their stomach to wombs has potential for new knowledge on reproduction that can be applied in new infertility treatments and therapies for women who have trouble carrying their pregnancy to term (7).

Besides interesting medical potential, the re-created species may, if released to wild, turn out to be environmentally or medically risky. Some fear that the re-created species may become vectors or reservoirs for viruses that can be harmful for other animals of human beings (7,15). Recent revival from Siberian permafrost of *Pithovirus sibericum*, the 30 000-year-old ancestral amoeba-infecting virus, pointed out such a likelihood (32). With respect to being potentially useful and risky at the same time, re-created organisms are very similar to many other bio-objects such as, for instance, genetically modified organisms. Another case, that conversely seems to propose more risks rather than benefits, is represented by the *Variola* virus stocks that are still preserved at two international centers, one in the USA and another in Russia, under the control of the World Health Organization, after the disease “smallpox” was declared eradicated in 1980 (33).

The most extreme case of de-extinction could be the re-creation of an extinct human species. Svante Pääbo, a renowned founder of paleogenetics, with his collaborators has provided a sequence of the genome of the *Homo Neanderthalis* (34). Even before this scientific breakthrough, there have been speculations about the adaptability of Neanderthals to the life of modern humans and about the moral acceptability to pursue this re-creation. Bioethically, the re-creation of Neanderthals constitutes a case that may be outright rejected. The re-creation of extinct human species by means of cross-species cloning is ethically analogous to a case of human cloning when a woman would be used as a surrogate mother in a scientifically uncertain experiment.

## CONCLUSION

De-extinction and its bioscientific applications offer useful insights to bio-objects and bio-objectification. De-extinction can be considered as a remarkable case study of an out-of-place bio-object and also a case study for bio-objectification of a whole species. Depending on how de-extinction is understood, it can be seen as a case of creating new bio-objects (new species) or as taking species under human control and thus turning them into bio-objects.
